# Supplementary material for: Does technological innovation in National Sustainable Development Agenda Innovation Demonstration Zones promote green development?—the case from Chengde City, China
Source: PLoS One. 2024 May 28;19(5):e0300315. doi: 10.1371/journal.pone.0300315 (PMC11132516; doi:10.1371/journal.pone.0300315)
Supplement: Attachment — Submitted filename: Response Letter.pdf [file pone.0300315.s002.pdf]

Response Letter for "Does Technological Innovation in National Sustainable Development Agenda Innovation Demonstration Zones Promote Green Development? --The Case from Chengde City, China"

Report of the Journal Requirements -- PONE-D-23-37873 /Zhang

Comments

1. Please ensure that your manuscript meets PLOS ONE's style requirements, including those for file naming. The PLOS ONE style templates can be found at https://journals.plos.org/plosone/s/file?id=wjVg/PLOSOne_formatting_sample_main_bc and https://journals.plos.org/plosone/s/file?id=ba62/PLOSOne_formatting_sample_title_aut

Response:

We are very grateful for the suggestions on the journal format. We have made changes to our manuscript based on the PLOS ONE style templates. And now our manuscript meets PLOS ONE's style requirements, including those for file naming.

2. Thank you for stating the following in the Acknowledgments Section of your manuscript: "This study was supported by the Key Public Project of Humanity and Social Sciences Research of Hebei Education Department (No. ZD202311), General Project of Soft Science Research of Hebei Provincial Department of Science and Technology (No.225576117D), Graduate Student Innovation Funding Project of Hebei Province (No. CXZZSS2023137)" We note that you have provided funding information that is not currently declared in your Funding Statement. However, funding information should not appear in the Acknowledgments section or other areas of your manuscript. We will only publish funding information present in the Funding Statement section of the online submission form. Please remove any

funding-related text from the manuscript and let us know how you would like to update your Funding Statement. Currently, your Funding Statement reads as follows: "the Key Public Project of Humanity and Social Sciences Research of Hebei Education Department (No. ZD202311); General Project of Soft Science Research of Hebei Provincial Department of Science and Technology (No.225576117D); Graduate Student Innovation Funding Project of Hebei Province (No. CXZZSS2023137)" Please include your amended statements within your cover letter; we will change the online submission form on your behalf.

Response:

Thanks for the suggestion that funding information should not appear in the Acknowledgments section or other areas of the manuscript. Therefore, we had removed the funding-related text from the manuscript. In addition, we would like to update our Funding Statement. And we would like the updated funding statement to be: General Project of Soft Science Research of Hebei Provincial Department of Science and Technology (No.225576117D); Graduate Student Innovation Funding Project of Hebei Province (No. CXZZSS2023137); the Key Public Project of Humanity and Social Sciences Research of Hebei Education Department (No. ZD202311). We have attached a revised statement to our cover letter; Thank you for making changes to the online submission form on our behalf.

Changes to the cover letter:

"In addition, our Funding Statement reads as follows: General Project of Soft Science Research of Hebei Provincial Department of Science and Technology (No.225576117D); Graduate Student Innovation Funding Project of Hebei Province (No. CXZZSS2023137); the Key Public Project of Humanity and Social Sciences Research of Hebei Education Department (No. ZD202311)." [Page 1]

3. Thank you for stating the following financial disclosure: "the Key Public Project of Humanity and Social Sciences Research of Hebei Education Department (No. ZD202311); General Project of Soft Science Research of Hebei Provincial

Department of Science and Technology (No.225576117D); Graduate Student Innovation Funding Project of Hebei Province (No. CXZZSS2023137)" Please state what role the funders took in the study. If the funders had no role, please state: "The funders had no role in study design, data collection and analysis, decision to publish, or preparation of the manuscript. If this statement is not correct you must amend it as needed. Please include this amended Role of Funder statement in your cover letter; we will change the online submission form on your behalf.

Response:

Thanks for your suggestion about illustrating the role of funders in this study. The funder of the "General Project of Soft Science Research of Hebei Provincial Department of Science and Technology (No.225576117D)" took the role of decision to publish in the study. The funder of the "Graduate Student Innovation Funding Project of Hebei Province (No. CXZZSS2023137)" took the role of data collection and analysis in the study. And the funder of the "the Key Public Project of Humanity and Social Sciences Research of Hebei Education Department (No. ZD202311)" took the role of data collection and analysis in the study. We have attached a revised statement to our cover letter; Thank you for making changes to the online submission form on our behalf.

Changes to the cover letter:

"And the roles of the funders are as follows: decision to publish in the study; data collection and analysis in the study; data collection and analysis in the study." [Page 1]

4. We note that you have stated that you will provide repository information for your data at acceptance. Should your manuscript be accepted for publication, we will hold it until you provide the relevant accession numbers or DOIs necessary to access your data. If you wish to make changes to your Data Availability statement, please describe these changes in your cover letter and we will update your Data Availability statement to reflect the information you provide.

Response:

Thanks for the suggestion about our Data Availability statement. And We do not need to change our data availability statement. Thank you for your attention.

5. PLOS requires an ORCID iD for the corresponding author in Editorial Manager on papers submitted after December 6th,2016. Please ensure that you have an ORCID iD and that it is validated in Editorial Manager. To do this, go to 'Update my Information'(in the upper left-hand corner of the main menu), and click on the Fetch/Validate link next to the ORCID field. This will take you to the ORCID site and allow you to create a new iD or authenticate a pre-existing iD in Editorial Manager. Please see the following video for instructions on linking an ORCID iD to your Editorial Manager account: https://www.youtube.com/watch?v=_xcclfuvtxQ

Response:

Thank you very much for the method to create ORCID iD in Editor Manager. It's very helpful to us. We have created an ORCID iD (0009-0009-9473-7807) and that it is validated in Editorial Manager.

6. Please ensure that you refer to Figure 1 in your text as, if accepted, production will need this reference to link the reader to the figure.

Response:

Thank you for your attention. In the manuscript, we have changed the information in Figure 1. And now we are sure that we refer to Figure 1 in our text.

7. If you are unable to complete any points that are requested in this email, please explain why in the 'Enter Comments' tab of the online submission form prior to re-submitting your manuscript.

Response:

Thank you for your attention. We have not presented written permission from the copyright holder to publish the figure. The reason is that: with regard to Figure 2, we directly use public welfare map with review numbers provided by the Ministry of

Natural Resources of the People's Republic of China as the base map. This base map is publicly available.

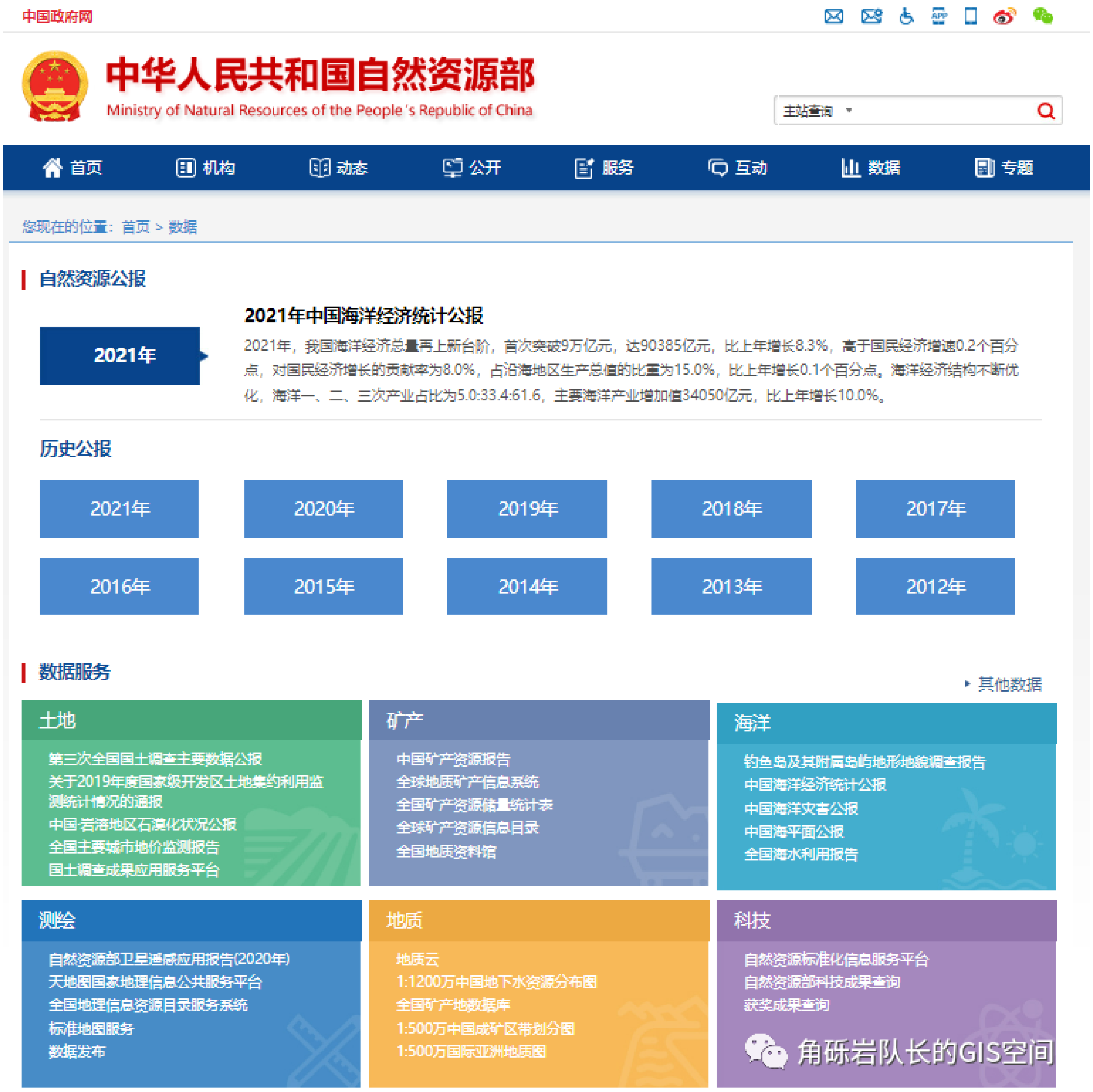

Fig1. The official website of the Ministry of Natural Resources of the People's Republic of China "Data" homepage

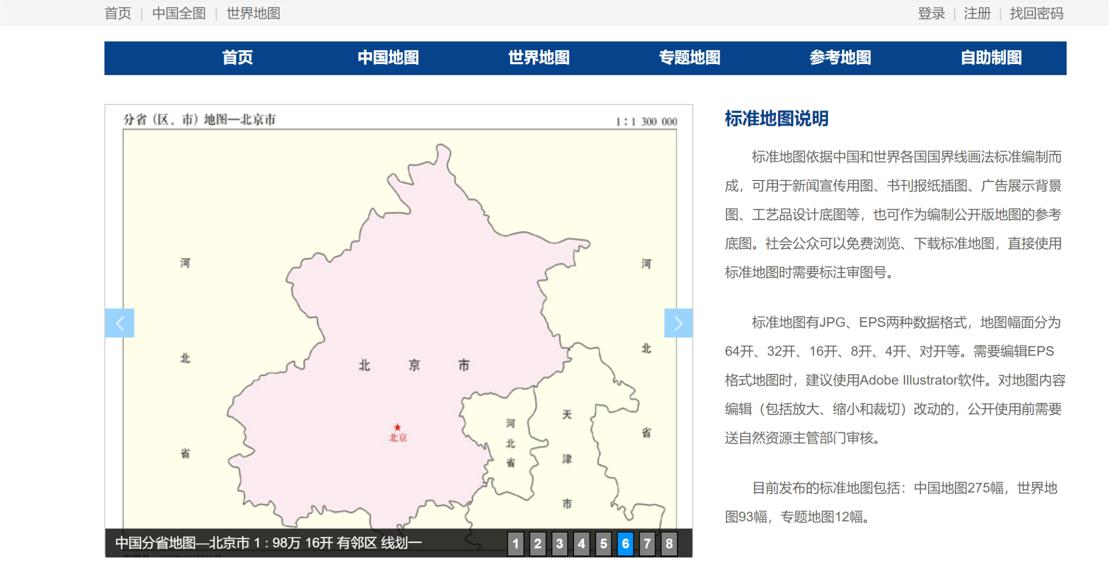

Fig2. The official website of the Ministry of Natural Resources of the People's Republic of China "Standard Map Use" homepage.

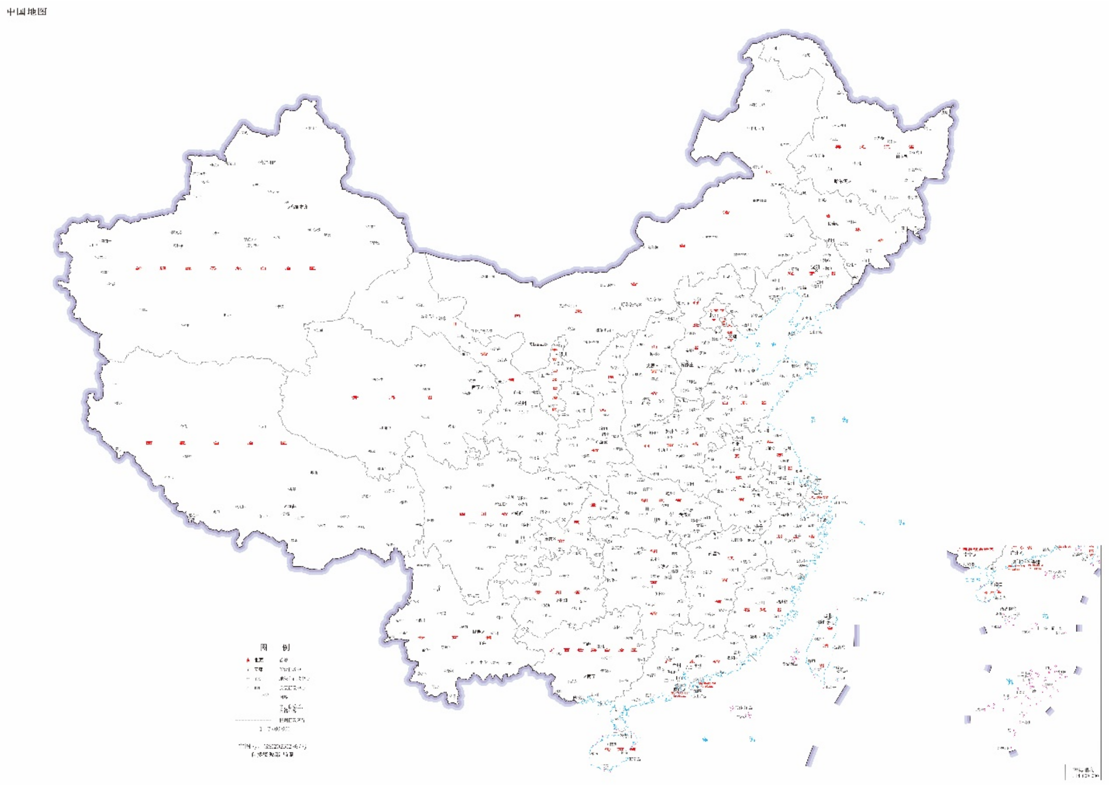

Fig3. Standard Map of China [Map Review Number: No. GS (2023) 2767].

Report of the First Reviewer -- PONE-D-23-37873 /Zhang

Comments

1. The introduction section is too long and needs to be presented more in terms of scholarly contributions.

Response:

We thank the reviewer's comments and suggestions. We agree with the reviewer that introduction section is too long and needs to be presented more in terms of scholarly contributions. We have revised the related discussions.

Changes to the manuscript:

Following the reviewer's suggestion, we have adjusted the structure of the introduction section and we have included the discussion about scholarly contributions. [Page 8, Line 157-165]

2. The chapter numbering in the whole text needs to be carefully proofread, especially in Mechanism and research hypotheses.

Response:

We thank the reviewer's suggestions. We agree with the reviewer that the chapter numbering in the whole text needs to be carefully proofread. We have revised the related chapter numbers.

Changes to the manuscript:

We have adjusted the chapter number in Mechanism and research hypotheses. [Page 8, Line 171; Page 9, Line 197-198; Page 11, Line 228-229]

3. Compared with Eq. (1), TI in Eq. (2) is not subscripted, and the squared term is incorrectly written as X.

Response:

We thank the reviewer's suggestions. We agree with the reviewer that Eq. (2) does have the writing error. And now Eq. (2) has been modified to Eq. (4).

Changes to the manuscript:

Following the reviewer's suggestion, we have corrected Eq. (2). Now Eq. (2) has been modified to Eq. (4). [Page 13, Line 267]

4. The selection of indicators needs to be clarified and a valid literature base needs to be added.

Response:

We appreciate the reviewers' suggestion. We've added the reasons and process for selecting the indicators. At the same time, literature has been added as a support. In addition, considering that the number of R&D personnel and the amount of R&D investment have a certain relationship with population density and local government fiscal revenue and expenditure, which may affect the research results, this paper uses the latter to measure the level of R&D investment.

Changes to the manuscript:

We have added the literature base to the selection of core explanatory and threshold variables. [Page 16, Line 327-331; Page 17, Line 339; Page 17, Line 340; Page 17, Line 342]

5. The selection of instrumental variables needs to be explained appropriately.

Response:

We appreciate the reviewers' suggestion. Following the reviewer's suggestion, we have included the explanation for the selection of instrumental variables. In line with the fundamental principles of constructing instrumental variables, a suitable instrument must satisfy two key conditions: first, it should exhibit a robust correlation with the endogenous variables, and second, it must be exogenous and independent of the error term. Referring to Zhang et al[1]., we adopt lagged one-period technological innovation as an instrumental variable, a common practice in the existing literature. This choice is grounded in the fact that technological innovation possesses a certain historical coherence, with the level of technological innovation in the previous period influencing the current period's technological innovation. Furthermore, after controlling for relevant demographic variables, economic factors, and incorporating city and year fixed

effects, it is established that technological innovation in the previous period does not directly impact the change in green development in the current period. Instead, its influence is mediated solely through the technological innovation of the current period, making it unrelated to the disturbance term of the current period. Consequently, the selected indicator logically fulfills both criteria for instrumental variables.

Changes to the manuscript:

In the revised version, with the help of helpful suggestions from reviewers, we have included the explanation for the selection of instrumental variables. [Page 22, Line 430-439]

6. The empirical results need to be compared with similar studies.

Response:

We appreciate the reviewer for the efforts to improve the quality of our manuscripts. We agree and accept the reviewer's comments. In the discussion section, we compare the empirical results with similar studies, and supplement the implications and shortcomings of this paper in terms of academic research.

Changes to the manuscript:

In the revised version, with the help of helpful suggestions from reviewers, we have added the comparison in the part of discussion. [Page 27, Line 526-552]

7. the conclusion section should be supplemented with the implications of this paper in terms of academic research

Response:

We appreciate the reviewers' suggestion. Following the reviewer's suggestion, we have included the discussion about the implications of this paper in terms of academic research.

Changes to the manuscript:

Building upon established findings linking technological innovation to green development, this study delves deeper into the ramifications of technological advancement within the context of current policies. Utilizing the Chengde National

Sustainable Development Agenda Innovation Demonstration Zone as a case study, it seeks to discern whether the impact of technological innovation varies under distinct conditions. The paper assesses the potential of R&D investment, industrial structure, and technological innovation to synergistically foster development and propel green initiatives, thereby addressing gaps in previous research. [Page 29, Line 570-578]

Report of the Second Reviewer -- PONE-D-23-37873 /Zhang

Comments

1. Regression Model Specification: Authors should clearly explain why square terms are introduced and elaborate on their theoretical basis in addressing the research question. The use of square terms should align with the theoretical framework in the research area. Otherwise, besides simply adding square terms, consider other more flexible nonlinear modeling methods such as spline regression, nonparametric estimation, or polynomial regression. Additionally, the use of square terms introduces challenges in interpreting nonlinear effects. The authors should provide more detailed explanations to ensure readers understand how square terms affect the dependent variable.

Response:

We appreciate the reviewers' suggestion about the regression model specification. We have added a discussion on whether there is a U-shaped relationship between technological innovation and green development in the mechanism study, and added quadratic terms of core explanatory variables to the empirical study for testing. Because the quadratic term of the core explanatory variable in the fixed-effect model did not pass the test, we did not proceed with the U-test to prove the authenticity of the U-shaped relationship.

We couldn't agree more strongly that in addition to simply adding square terms, there are other, more flexible nonlinear modeling methods to consider, such as spline regression, nonparametric estimation, or polynomial regression. However, based on the previous research on whether the relationship between technological innovation and green development is a U-shaped relationship, this paper chooses to add a square term.

Changes to the manuscript:

"However, scholars continue to debate whether the relationship between technological innovation and green development is linear. It is not universally true that higher levels of technological innovation always result in more significant promotion of green development. When the level of technological innovation is low, the efficiency

of resource and energy utilization is low; along with the enhancement of technological innovation, advanced cleaner and other production technologies have been widely used, promoting the reduction of resource and energy consumption and emissions of three wastes. Therefore, there may be a U-shaped relationship between technological innovation and green development." [Page 9, Line 185-193]

"Among them, column (1) presents the regression result with all control variables included. In order to test whether there is a U-shaped relationship between technological innovation and green development, the quadratic terms of core explanatory variables are added to column (1), and the regression results are shown in column (2)." [Page 19, Line 386-390]

2. Endogeneity Issues: I am concerned about the endogeneity issues in this article, such as

   a) Omitted Variables: Problem: e.g. The model may not have considered the impact of urban planning policies, which could be a crucial omitted variable. Explanation: If urban planning policies affect both technological innovation and urban green development and are not considered in the model, the model might underestimate the actual impact of technological innovation on urban development.

Response:

We thank the reviewer's comments. We agree and accept the reviewer's comments. There are two major endogenous problems in identifying the impact of technological innovation on green development. The first is the problem of missing variables. In the benchmark regression model, multiple factors such as the scale of fiscal expenditure, population density and urbanization rate that affect green development should be controlled as much as possible, and the fixed effects of regions and years should be controlled to reduce the endogeneity caused by the omission of variables. However, technological innovation may still be affected by other uncontrolled third-party factors or policies, which may also affect green development. As the reviewers mentioned, the model may not have considered the impact of urban planning policies, which could be a crucial omitted variable.

In order to promote sustainable development, Chengde has formulated the *Chengde Sustainable Development Plan (2018-2030) (Revised in 2022)* and the *Chengde National Sustainable Development Agenda Innovation Demonstration Zone Construction Plan (2021-2025)* in recent years. It has played an important role in the construction of the demonstration area. However, due to the difficulty of obtaining data on urban planning policies in the county data used in this paper, the impact of urban planning policies on technological innovation and green development is not considered.

b) Bidirectional Causation:

Problem: The model may not have considered the feedback effects of technological innovation on green development.

Explanation: If an increase in technological innovation leads to more green development, and higher levels of green development, in turn, encourage more technological innovation, the model might underestimate the true relationship between technological innovation and green development. In this case, the model might need to more comprehensively consider this mutual influence to accurately capture the causation between the two.

Response:

We appreciate the reviewer for the efforts to improve the quality of our manuscripts. We agree and accept the reviewer's comments. It is true that the model does not take into account the feedback effect of technological innovation on green development. If an increase in technological innovation leads to more green development, and higher levels of green development, in turn, encourage more technological innovation. Therefore, we examine the endogeneity by examining the impact of green development on technological innovation.

Changes to the manuscript:

"Considering the feedback effects of technological innovation on green development, i.e., if an increase in technological innovation leads to more green development, and higher levels of green development, in turn, encourage more technological innovation. This study examines the endogeneity by examining the impact

of green development on technological innovation. The results show that green development has a significant positive impact on technological innovation." [Page 21, Line 425-431]

3. Instrumental Variable Issues:

   If the lagged one-period variables used as instrumental variables are endogenous (i.e., influenced by common unobserved factors), the instrumental variable may still be subject to endogeneity issues, rendering it ineffective in addressing endogeneity. Additionally, lagged variables may be unstable, especially in the presence of external shocks or structural changes. This may cause the instrumental variable's effects to vary over time, making it ineffective during different time periods, and you assume structural changes indeed exist. Besides the instrumental variable method, have you considered other methods to address endogeneity? Please discuss the reasons for choosing the instrumental variable method and, where possible, consider comparing it with other endogeneity handling methods.

Response:

We appreciate the reviewers' suggestion. Following the reviewer's suggestion, we have included the explanation for the selection of instrumental variables. In line with the fundamental principles of constructing instrumental variables, a suitable instrument must satisfy two key conditions: first, it should exhibit a robust correlation with the endogenous variables, and second, it must be exogenous and independent of the error term. Referring to Zhang et al., we adopt lagged one-period technological innovation as an instrumental variable, a common practice in the existing literature. This choice is grounded in the fact that technological innovation possesses a certain historical coherence, with the level of technological innovation in the previous period influencing the current period's technological innovation. Furthermore, after controlling for relevant demographic variables, economic factors, and incorporating city and year fixed effects, it is established that technological innovation in the previous period does not directly impact the change in green development in the current period. Instead, its influence is mediated solely through the technological innovation of the current period,

making it unrelated to the disturbance term of the current period. Consequently, the selected indicator logically fulfills both criteria for instrumental variables.

Besides the instrumental variable method, we have considered other methods to address endogeneity, such as DID-GMM model and SYS-GMM model. The SYS-GMM model stands out as a superior estimation method, proficiently addressing issues such as autocorrelation, heteroskedasticity, endogeneity, and weak instrumental variables. This approach ensures the production of unbiased, efficient, and consistent estimation results. Notably, the SYS-GMM model adeptly tackles the challenge of endogeneity between the explained variables and their lagged counterparts, mitigating the risks of biased and inconsistent model analysis. In examining the impact of technological innovation on regional green development, we opt for the SYS-GMM model. Here, regional green development (GD) serves as the explanatory variable, incorporating the lagged 1-period green development into the model. The core explanatory variable is technological innovation (TI). This model selection meets the condition of having a dynamic explanatory variable and not all explanatory variables strictly being exogenous. Moreover, it includes controls for individual and time double fixed effects. The results of the SYS-GMM model regression are shown in the table below.

Table. SYS-GMM test

Variables	GMM teat
TI	0.0499^{**}(0.0225)
Control	YES
Observation	77
AR (1)	0.042
AR (2)	0.137
Hansen	1.000

The significance of the core explanatory variables remained consistent, and the p-values for both the Hansen and AR(2) test statistics exceeded 10%. This lack of statistical significance prevents the rejection of the original hypothesis, indicating the

absence of autocorrelation. Consequently, the rationale behind the establishment of the SYS-GMM model is justified, and the study's findings are validated as robust.

Both the system GMM model and the instrumental variables method are pivotal techniques for addressing endogeneity issues. However, the system GMM model exhibits several drawbacks in comparison to the instrumental variables method: (1) the necessity to choose the lag order in the system GMM model, with improper selection potentially introducing uncertainty into the results; (2) the system GMM model imposes higher demands for a substantial number of temporal observations and individual observation data. In scenarios with sparse data, the model's effectiveness may be constrained. Consequently, in this paper, the instrumental variable method is chosen as the preferred approach.

Changes to the manuscript:

In the revised version, with the help of helpful suggestions from reviewers, we have included the explanation for the selection of instrumental variables. [Page 22, Line 430-440]

4. Threshold Effects:

   In the manuscript, there needs to be a clearer expression of the theoretical basis for threshold effects. Explain why the study assumes the existence of threshold effects and their rationale in addressing the research question. Although threshold values are mentioned in the text, there seems to be no detailed discussion on how this threshold value is chosen. In threshold effect studies, the choice of the threshold is crucial as it directly influences the interpretation of study results. A thorough theoretical and empirical argumentation is needed for the selection of the threshold value, and consideration should be given to whether there are multiple potential threshold values. When explaining threshold effects, ensure that the results are interpretable to readers. Clearly state the significance of threshold effects for the research question and why threshold effects make theoretical sense. While statistical significance is important, also pay attention to substantive significance. In threshold effect studies, ensure that the threshold effects are not only statistically significant but also substantively meaningful in practice.

Response:

We thank the reviewer's comments. We agree and accept the reviewer's comments. In the section of **materials and methods**, we add mathematical models and theoretical foundations in order to better explain the threshold effect. At the same time, we explain these three questions: why the study hypothesizes a threshold effect, why the threshold effect is important to the research question, and why the threshold effect is theoretically meaningful.

In the section of **non-linear effect of R&D investments on the impact of technological innovation on green development**, we utilize GD as the green development index, TI as the innovation output index, and RD as the industrial structure upgrading element, then propose a breakpoint model as follows:

$$\begin{cases} GD = \alpha_1 TI + \varepsilon,\ RD < Threshold_1 \\ GD = \alpha_2 TI + \varepsilon,\ Threshold_1 \leq RD \leq Threshold_2 \\ GD = \alpha_3 TI + \varepsilon,\ RD > Threshold_2 \end{cases}$$

When R&D investment is insufficient or excessive, the effect of technological innovation on green development will be weakened, i.e. $\alpha_1 < \alpha_2, \alpha_3 < \alpha_2$. Therefore, only when R&D investments and technological innovation form a benign interactive relationship, can the promotion effect of both on green development be maximized.

In the section of **non-linear effect of industrial structure upgrading on the impact of technological innovation on green development**, we utilize GD as the green development index, TI as the innovation output index, and STR as the industrial structure upgrading element, then propose a breakpoint model as follows:

$$\begin{cases} GD = \beta_1 TI + \varepsilon, \quad STR < Threshold \\ GD = \beta_2 TI + \varepsilon, \quad STR \geq Threshold \end{cases}$$

Based on the above theoretical argument on the choice of threshold, this paper conducts an empirical demonstration and considers that there are multiple potential thresholds that should be considered, but the threshold level is not reached due to the low level of R&D investment in Chengde city.

Changes to the manuscript:

"First, in cases where R&D investment falls short, the anticipated benefits may remain unrealized. In terms of innovation theory interpretation, it is acknowledged that

innovation activities necessitate substantial R&D investment. However, a simplistic linear relationship does not align with the fundamental attributes of R&D endeavors. Throughout the innovation process, a significant infusion of talent, capital, and other R&D resources occurs in the early stages. It is only through persistent accumulation that the level of technological innovation can be enhanced." [Page 10, Line 201-224]

"It does not appear that technological innovation has an inhibitory effect on green development due to excessive R&D investment. This can be mainly explained by the fact that the level of R&D investment in Chengde is low and may not have reached the level of the threshold." [Page 26, Line 502-505]

Report of the Third Reviewer -- PONE-D-23-37873 /Zhang

Comments

The authors take Chengde City as the case study to investigate the impact of technological innovation on green development, additionally, taking the Research and development investments as the threshold variable. Overall, the topic is interesting. However, there are some issues, from my point of view, deserve to be treated before publication.

Response:

We are very grateful to the reviewer for his/her positive comments and insightful summaries of our work.

1. I would suggest a bit rework of the paper in the introduction. In its current version, the paper spends too much ink on the description of national policies, but the elaboration of the relationship between technological innovation, green development, R&D investment, and industrial structure upgrading is a bit abrupt and lacks corresponding references, which needs to be further improved.

Response:

We thank the reviewer’s comments. We agree and accept the reviewer's comments. We have taken a bit rework of the paper in the introduction. We reduced the description of national policies and elaborated on the relationship between technological innovation, green development, R&D investment, and industrial structure upgrading.

The structure of the introduction is as follows: In the first paragraph of the introduction, we use the change in the definition of sustainable development to highlight the importance and necessity of the implementation of the SDGs. In the second paragraph of the introduction, we describe the process of sustainable development in China and internationally (Fig 1). In the third paragraph of the introduction, we raise the scientific questions that this paper will explore. In the paragraph of 4, 5, 6 and 7, we demonstrate the relationship between technological innovation and green development. In the sixth paragraph of the introduction. In the

paragraph of 8, we demonstrate the relationship between technological innovation, green development, R&D investment, and industrial structure upgrading.

Changes to the manuscript:

"The definition of sustainable development has been broadened from "meeting the needs of the present without compromising the ability of future generations to meet their own needs" to "inclusive well-being" – the aggregate quality of life for all people, everywhere, now and in the future-does not decline with time"[2, 3]. In September 2015, the United Nations Development Summit adopted the 2030 Agenda for Sustainable Development, which established global Sustainable Development Goals (SDGs) that provide specific targets for enhancing inclusive well-being[4]."

"A number of organizations and countries have incorporated the 2030 Agenda into their national development plans [5, 6]. At the same time, scholars worldwide have conducted extensive research on the implementation of SDGs, particularly on SDG 3[7, 8], SDG 4[9, 10], SDG 6[11], and SDG 12[12, 13]. China, along with the international community, is actively advancing the implementation of a global sustainable development strategy (Fig 1). Since 2016, the State Council of China initiated the establishment of 11 National Sustainable Development Agenda Innovation Demonstration Zones (NSDAIDZs) (Fig 2). Each demonstration area has its specific implementation content and characteristics." [Page 3, Line 48-63]

"The relationship between technological innovation, industrial structure, R&D investment and green development is also an area of continuing scholarly interest. Song[14] argued that the shift to green technology-based production is imperative for achieving sustainable development, especially in key industries. The efficiency of technological innovation, and the advanced industrial structure are all conducive to the improvement of green development in manufacturing industry[15]. If there is an increase in the spatial imbalance of green technological innovation, industrial structure rationalization, and industrial structure advancement, the gap between carbon emission efficiency will be increased, and regional sustainable development will be adversely affected[16]." [Page 6, Line 118-138]

2. The research framework, including R&D investments, industrial structure, technological innovation and green development, needs to be further clarified. Technological innovation and R&D investment are very broad concepts that involve various industries, and probably only some of them are related to green development. Therefore, I am wondering whether it is possible to change these variables to be more relevant to green development.

Response:

Thank you for your valuable feedback and suggestions regarding our study. We appreciate your attention to detail and have taken your comments into consideration. We agree with you that technological innovation and R&D investment are very broad concepts that involve various industries, and probably only some of them are related to green development. Therefore, we have considered using two variables that are more related to green development, environmental R&D investment and green technology innovation, to characterize R&D investment and technological innovation. However, due to the difficulty of obtaining county-level data and the serious lack of cross-year data, the variables used in this paper were finally selected.

3. The authors argue that the work is novel because it uses Chengde City, the NSDAIDZs, as a case study to investigate the impact of technological innovation on green development, which has previously been overlooked.

Response:

We are very grateful to the reviewer for his/her interest in the work we do.

I have two questions about this.

First, Chengde City was identified as the NSDAIDZs in May 2019, but the panel data in this paper is selected from 2014 to 2020.It would be better if the study period could be extended to compare the changes in this effect before and after the establishment of the NSDAIDZs. Second, the theme of Chengde being identified as the NSDAIDZs is closely related to its water resources, and these can be discussed. Third, is it possible to have a map to illustrate the location of Chengde in China?

Response:

We appreciate the reviewers' suggestion. First, we agree with the reviewer's suggestion that it would be better if the study period could be extended to compare the changes in this effect before and after the establishment of the NSDAIDZs. The panel data in this paper is selected from 2014 to 2020, because county data is not currently available after 2020, so we are also looking at the dynamics of county data after 2020. We will supplement the data as soon as the county data from 2020 onwards is available. Second, the theme of Chengde being identified as NSDAIDZ is closely related to its water resources, which is another topic we will study next. Third, we have used a map to illustrate the location of Chengde and other demonstration areas in China (Fig 2). The topic of Chengde being identified as NSDAIDZ is closely related to its water resources,

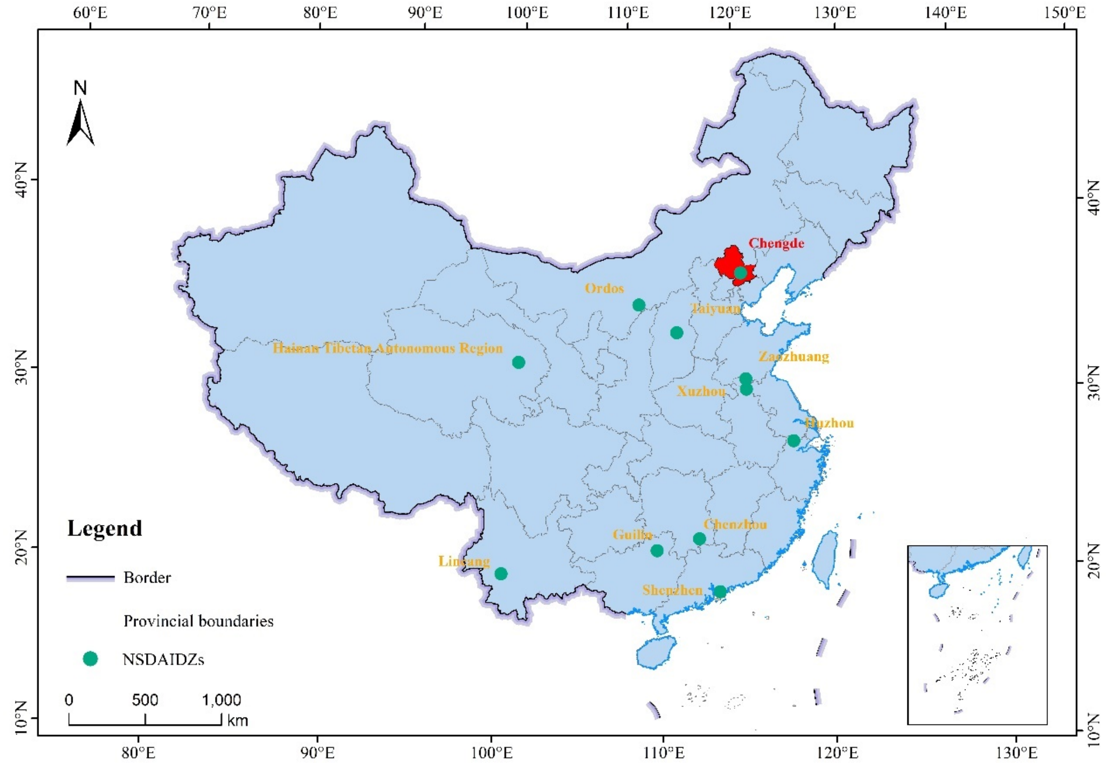

Fig 2. Overview of the distribution of National Sustainable Development Agenda Innovation Demonstration Zones

Report of the fourth Reviewer -- PONE-D-23-37873 /Zhang

Comments

This article takes Chengde city as an example to examine the quantitative impact of technological innovation on green development in the National Sustainable Development Agenda Innovation Demonstration Zones (NSDAIDZs), and the topic has certain practical significance.

Response:

We are very grateful to the reviewer for his/her positive comments of our work.

However, the article has the following obvious problems:

(1) The innovation is not obvious, and there is already a lot of research on the impact of technological innovation on green development, and the difference between this article and existing research is not significant.

Response:

We appreciate the reviewers' suggestion. In the discussion section, we compare the empirical results with similar studies, and supplement the implications and shortcomings of this paper in terms of academic research.

Changes to the manuscript:

In the revised version, with the help of helpful suggestions from reviewers, we have added the comparison in the part of discussion. [Page 27-29, Line 526-564]

(2) The conclusion of the article can only indicate that technological innovation in Chengde has promoted green development, but cannot indicate that this impact is caused by NSDAIDZs policy. In fact, the empirical research in this article cannot exclude the influence of other policies or factors.

Response:

Thank you for the reviewer's valuable feedback regarding our study. Following the reviewer's suggestion, we have made two changes in this paper: one is to consider that the implementation of the demonstration zone policy in H1 has a stronger role in

promoting technological innovation to green development, so the mechanism diagram has been modified, and the other is to carry out a phased regression in the benchmark regression to increase the comparison of the impact of technological innovation on green development before and after the implementation of the policy.

Changes to the manuscript:

Fig 3. [Page 12, Line 261-262]

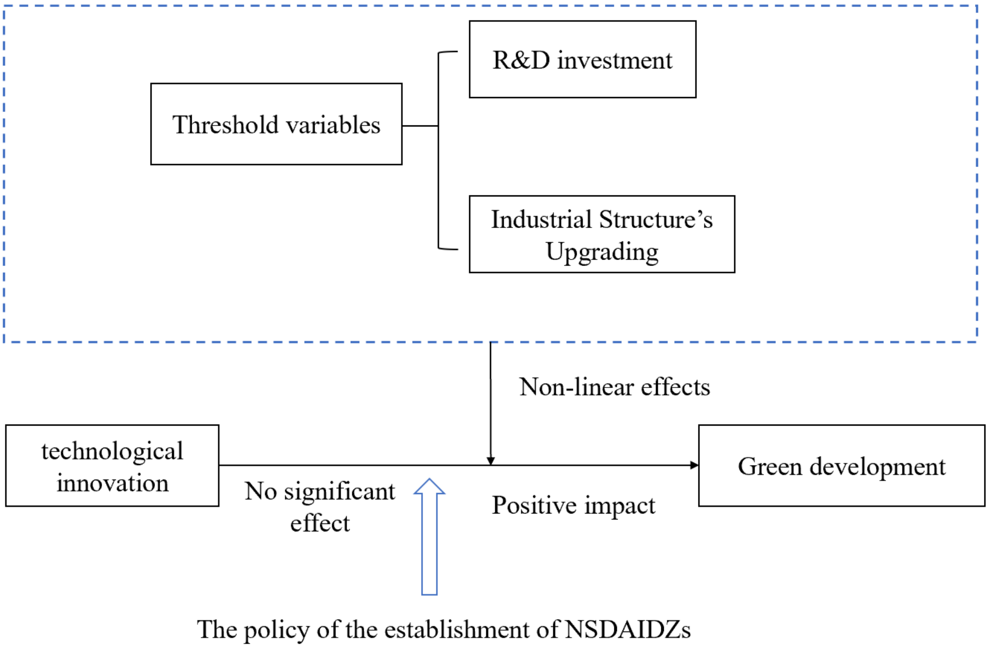

Fig 3. Research framework of the impact of technological innovation in National Sustainable Development Agenda Innovation Demonstration Zones on green development.

"The temporal division into pre and post-establishment phases of the National Sustainable Development Agenda Innovation Demonstration Zone allows for separate regression analyses, with the results presented in columns (3) and (4). Following the decision to establish the National Sustainable Development Agenda Innovation Demonstration Zone in Chengde City, the influence of technological innovation on green development transitions from a negative impact to a notably positive one. This observation suggests that the implementation of the National Sustainable Development Agenda Innovation Demonstration Zone policy can substantially amplify the positive catalytic effect of technological innovation on promoting green development." [Page 20, Line 396-405]

(3) The theoretical mechanism is too generalized. Even without NSDAIDZs policies, technological innovation can still promote green development, and the conclusion is obvious. The article lacks in-depth analysis of the specific implementation content and characteristics of China's NSDAIDZs policy.

Response:

We agree with the reviewers that technological innovation can still promote green development even without policies. But for Chengde, the conclusion may be different. According to the benchmark regression results, technological innovation in Chengde cannot promote green development well when the policy is not implemented, which may be due to the low level of technological innovation.

Fig 1 depicts the process of sustainable development in China and internationally, and the predecessor of the Chengde National Sustainable Development Agenda Innovation Demonstration Zone was the Chengde National Sustainable Development Experimental Zone. The big difference between the National Sustainable Development Agenda Innovation Demonstration Zone and the National Sustainable Development Experimental Zone is that the National Sustainable Development Agenda Innovation Demonstration Zone conforms to the development of the times and highlights the use of innovation to solve bottlenecks in the process of sustainable development, which is also one of the original intentions of our discussion on technological innovation and green development.

Changes to the manuscript:

"Numerous scholars have empirically demonstrated the driving effect of technological innovation on green development, yet little attention has been paid to assessing whether this impact varies across different conditions. Utilizing Chengde National Sustainable Development Agenda Innovation Demonstration Zone as a case study, this research integrates the triad of R&D investments, industrial structure, and technological innovation into a comprehensive analytical framework. The objective is to explore whether these elements collectively form the capacity to coordinate development and foster green development. The study concludes that technological innovation indeed exhibits a catalytic effect on green development, aligning with prior

scholarly findings. Furthermore, it reveals a pronounced driving effect of technological innovation on green development following the implementation of the National Sustainable Development Agenda Innovation Demonstration Zone policy. Consequently, the anticipated "rebound effect" triggered by technological innovation is not observed. Considering the feedback loop of technological innovation on green development—where increased technological innovation leads to more green development, and higher levels of green development, in turn, encourage further technological innovation—the empirical results indicate a reverse causal relationship between technological innovation and green development." [Page 27-28, Line 526-544]

(4) The article only uses data from 11 districts in Chengde City from 2014 to 2020, with a small sample size. The empirical research methods and robustness tests are too simple, and the basis is insufficient, which will affect the reliability of the conclusions.

Response:

Thank you for the reviewer's valuable feedback regarding our study. We agree with the reviewer's comments. Firstly, the sample size is small, because there are some limitations in obtaining data in the county. We are also actively contacting relevant departments and collecting data from other demonstration areas as well as from 2020 onwards. Second, the empirical research method and robustness test are too simple. We added the test of reverse causality problem to the endogeneity test, and we also used methods other than instrumental variables method, and systematic GMM model to deal with the endogeneity problem.

(5) The conclusions obtained have no effect on how to further improve NSDAIDZs, and the targeted and actionable policy recommendations are not strong.

Response:

We appreciate the reviewer' comment. We agree with the reviewer that we need to explain the implications of the conclusions for further improving NSDAIDZs, and make targeted and actionable policy recommendations.

Utilizing the Chengde National Sustainable Development Agenda Innovation

Demonstration Zone as a case study, we try to reveal the impact of technological innovation on green development in this policy context. We found that there is a noteworthy disparity in the impact of technological innovation on green development before and after the establishment of National Sustainable Development Agenda Innovation Demonstration Zone.

According to the conclusion, the following policy suggestions are put forward: (1) Reinforce the impact of policy support, bolster the capacity for technological innovation, and ignite the vibrancy of innovation. (2) Attach importance to the role of technological innovation in environmental governance, improving the quality of growth, and ecological protection. (3) Proactively nurture the positive interaction among R&D investment, industrial structure, technological innovation, and green development.

Changes to the manuscript:

"The establishment of NSDAIDZs is the major initiative of China's Country Program for the Implementation of the 2030 Agenda for Sustainable Development, and also provides experience and reference for other countries and regions in the world with similar sustainable development problems."

"(1) Reinforce the impact of policy support, bolster the capacity for technological innovation, and ignite the vibrancy of innovation. Aligned with the directives of the National Sustainable Development Agenda Innovation Demonstration Zone, we will rigorously execute national and provincial science and technology innovation policies. On one hand, we aim to steer enterprises towards escalating R&D investments, thereby stimulating their innovation capabilities. On the other hand, we seek to encourage social capital to actively engage in and support corporate research initiatives. This multifaceted approach aims to comprehensively facilitate the interconnected development of science, technology, industry, and finance. (3) Proactively nurture the positive interaction among R&D investment, industrial structure, technological innovation, and green development. Thoughtfully elevating the level of R&D investment and optimizing industrial structure can synergistically complement sustainable development support policies, thereby reinforcing the role of technological innovation in advancing green development."[Page 31-32, Line 594-596; Line 600-608; Line 621-

625]

Reference

[1] ZHANG S, XU M, ZHU Y, et al. Technological Innovation,Organizational Resilience and High-quality Development of Manufacturing Enterprises [J]. Science& Technology Progress and Policy, 2023, 40(13): 81-92.

[2] CASSEN R H. Our common future: report of the World Commission on Environment and Development [J]. International Affairs, 1987, 64(1): 126-.

[3] DASGUPTA P. Human Well-Being and the Natural Environment [M]. Oxford University Press, 2001.

[4] RONZON T, SANJUáN A I. Friends or foes? A compatibility assessment of bioeconomy-related Sustainable Development Goals for European policy coherence [J]. Journal of Cleaner Production, 2020, 254: 119832.

[5] XIE H, WEN J, CHOI Y. How the SDGs are implemented in China——A comparative study based on the perspective of policy instruments [J]. Journal of Cleaner Production, 2021, 291: 125937.

[6] ZHANG C. ow the SDGs are implemented in China——A comparative study based on the perspective of policy instruments [J]. Crossroads: Southeast Asian Studies, 2018, (4): 56-66.

[7] FULDAUER L I, IVES M C, ADSHEAD D, et al. Participatory planning of the future of waste management in small island developing states to deliver on the Sustainable Development Goals [J]. Journal of Cleaner Production, 2019, 223: 147-62.

[8] ZHAO Y, TAN Y, FENG S. Does reducing air pollution improve the progress of sustainable development in China? [J]. Journal of Cleaner Production, 2020, 272: 122759.

[9] LEAL FILHO W, SHIEL C, PAçO A, et al. Sustainable Development Goals and sustainability teaching at universities: Falling behind or getting ahead of the pack? [J]. Journal of Cleaner Production, 2019, 232: 285-94.

[10] LEAL FILHO W, FRANKENBERGER F, SALVIA A L, et al. A framework for the implementation of the Sustainable Development Goals in university programmes [J]. Journal of Cleaner Production, 2021, 299: 126915.

[11] HO L, ALONSO A, EURIE FORIO M A, et al. Water research in support of the Sustainable Development Goal 6: A case study in Belgium [J]. Journal of Cleaner Production, 2020, 277: 124082.

[12] KHALED R, ALI H, MOHAMED E K A. The Sustainable Development Goals and corporate sustainability performance: Mapping, extent and determinants [J]. Journal of Cleaner Production, 2021, 311: 127599.

[13] YAGI M, KOKUBU K. A framework of sustainable consumption and production from the production perspective: Application to Thailand and Vietnam [J]. Journal of Cleaner Production, 2020, 276: 124160.

[14] SONG Y, ZHANG Z, SAHUT J-M, et al. Incentivizing green technology innovation to confront sustainable development [J]. Technovation, 2023, 126: 102788.

[15] CHENG M, WEN Z, YANG S. The driving effect of technological innovation on green development: dynamic efficiency spatial variation [J]. Environmental Science and Pollution

Research, 2022, 29(56): 84562-80.

[16] SUN Z, SUN Y, LIU H, et al. Impact of spatial imbalance of green technological innovation and industrial structure upgradation on the urban carbon emission efficiency gap [J]. Stochastic Environmental Research and Risk Assessment, 2023, 37(6): 2305-25.
